# Effects of Demographic Factors and Comorbidities on SAR-CoV-2 (Coronavirus) Severity in Southern New Mexico

**DOI:** 10.1016/j.focus.2025.100467

**Published:** 2025-11-05

**Authors:** Naheed Shahbaz, Robert Goldsteen, Soyoung Jeon, Rachel Brown, Andrew Issa

**Affiliations:** 1 Center for Healthcare Equity in Kidney Disease (CHEK-D), Department of Internal Medicine, University of New Mexico, Albuquerque, New Mexico; 2 Burrell College of Osteopathic Medicine, Las Cruces, New Mexico; 3 Department of Economics, Applied Statistics and International Business, College of Business, New Mexico State University, Las Cruces, New Mexico

**Keywords:** COVID-19, SARS-CoV-2, health disparities, hospitalization risk, comorbidities, U.S.-Mexico border

## Abstract

**Introduction:**

Globally, COVID-19 has caused widespread morbidity and mortality, with comorbidities and demographic disparities shaping risk of severe illness. Although much focus has been on high-population areas and areas with deep public health responses, relatively few studies have examined the Southwestern U.S., particularly the interborder region with Mexico. This study investigates how the COVID-19 pandemic affected patients with common comorbid conditions and examines and compares outcomes in border counties and selected nonborder counties in southern New Mexico.

**Methods:**

The data set of COVID-19–positive patients (N=93,586) was collected from the New Mexico Department of Health (June 2020–December 2022). This study examined the associations between demographic characteristics; underlying comorbidities; and COVID-19 outcomes, including hospitalization and death.

**Results:**

The patients assessed were more likely to be female (54.8%), Hispanic (48.5%), and border county residents (95.7%). Of all patients, 4,445 (4.8%) were hospitalized, and 1,517 (1.6%) died. Hispanics were associated with higher deaths (OR=1.60; 95% CI=1.28, 1.99), whereas hospitalization did not differ by ethnicity (*p*=0.072). Nonborder resident status (*p*<0.001) and pre-existing conditions were significantly associated with increased risk of hospitalization (OR=4.87; 95% CI=3.99, 6.00) and mortality (OR=2.92; 95% CI=1.72, 5.33). All comorbidities except psychiatric conditions were risk factors for severity and all-cause mortality (all *p*<0.001).

**Conclusions:**

This study identified key demographic and clinical factors associated with SARS-CoV-2 outcomes in the New Mexico–Mexico border region. Older age, male adults, residence in nonborder counties, and pre-existing medical conditions were significant predictors of hospitalization and mortality, with several comorbidities showing particularly strong associations. Unlike other studies, in the New Mexico/Mexico region, the greater access to screening and medical services in border zones, given the proximity to 2 larger cities (Las Cruces, NM and El Paso, TX), resulted in improved outcomes than in less well-resourced counties in New Mexico. State departments of health should target state regions with less access to nearby populated areas for their efforts to mitigate these disparities and improve health outcomes.

## Highlights

• The study analyzed COVID-19 outcomes among 93,586 adults in southern New Mexico.
• It identified age, male sex, and comorbidities as main predictors of mortality.
• Nonborder residents faced higher hospitalization and death risks.
• Hispanic ethnicity was linked to higher death but not hospitalization odds.
• Border proximity to Las Cruces and El Paso improved patient outcomes.

## INTRODUCTION

Severe acute respiratory syndrome coronavirus 2 (SARS-CoV-2) was declared a global pandemic by the WHO on 11 March 2020 owing to its rapid spread and pathogenicity.^1^ Since the first confirmed case of coronavirus disease 2019 (COVID-19) in the U.S. was reported on January 20, 2020, the SARS-CoV-2 deaths in the U.S. have reached 1.2 million through February 11, 2024.^2^ As of March 5, 2025, there had been 777,504,802 confirmed SARS-CoV-2 cases and 7,089,979 deaths reported globally. The U.S. leads in both SARS-COV-2 cases and deaths (1.2 million), whereas Mexico ranks fifth in fatalities (335 thousand).^3^ The COVID-19 death rate in New Mexico (NM) is approximately 435.66 deaths per 100,000 population compared with that in the U.S. with about 368 deaths per 100,000 population (WHO). NM experienced an increased morbidity and mortality during SARS-CoV-2 period and had ranked 43^rd^ in deaths from preventable and treatable causes between 2019 and 2021 during the COVID-19 pandemic. There was a greater than 35% increase in avoidable mortality rates over this period.^4^ Previous research has identified that Latinos in the U.S. are 3 times more likely to be affected by COVID-19 and 2 times more likely to die from the disease.^5^ These figures underscore the comparatively higher burden of cases and mortality in NM and the need to examine the impact of such pandemics on the Latino population in particular.^3^

Understanding the relationship between comorbidities, demographic factors, and SARS-COV-2 outcomes is crucial for identifying vulnerable populations and improving future health department responses and overall patient care strategies to apply toward future pandemics. Knowing that SARS-CoV-2 poses global risk, causing significant illness, morbidity, and mortality,^6^ the authors wished to determine whether there was equivalent heightened risk and severity of SARS-CoV-2 in a cohort of NM patients, including Hispanic patients, those with diabetes, and those of older age, compared with national data.^7^^,^^8^ A retrospective study showed that middle-aged and elderly patients with SARS-CoV-2 are more susceptible to illness and have a higher likelihood of intensive care unit admissions and increased mortality rate.^9^ In addition, disparities are evident across multiple factors, such as sex, age, ethnicity, occupation, SES, smoking habits, and underlying medical conditions.^10^

**S**tudies in China have shown hypertension to be an important risk factor for mortality among intensive care unit patients with COVID-19.^11^ Furthermore, Chinese data showed that older adults with multiple comorbidities had higher risk of severe outcomes and death.^12^ In the U.S., surveillance data indicated that most hospitalized patients had underlying comorbidities, with obesity, hypertension, and diabetes being the most common.^13^

Similar findings have been reported in other regions worldwide, including more developed and underdeveloped countries. In Italy, almost half of COVID-19 deaths occurred among patients with 3 or more chronic conditions, underscoring the cumulative effect of multimorbidity.^14^ In Brazil, socioeconomic inequalities compounded risks, with disadvantaged populations experiencing higher hospitalization and death rates independent of age and comorbidities.^15^ In Mexico, national surveillance data identified obesity, diabetes, and hypertension as leading predictors of COVID-19 mortality.^16^ In the Middle East, studies in Saudi Arabia and Iran highlighted diabetes and obesity as the most consistent predictors of severe outcomes and death, especially in younger adults.^17^^,^^18^ In South Africa, analyses in a high HIV/tuberculosis prevalence setting revealed that these conditions significantly increased the risk of hospitalization and in-hospital mortality, alongside noncommunicable diseases.^19^^,^^20^ Collectively, these studies show that although the mix of risks differs by region, comorbidities and social disadvantage consistently worsen COVID-19 outcomes.

This study aims to address this gap by investigating the impact of the SARS-CoV-2 pandemic on patients with common comorbidity conditions across 8 counties in southern NM. Specifically, the authors aim to examine the associations between demographic characteristics; underlying comorbidities; and SARS-CoV-2 demographics, comorbidities, and health outcomes near the U.S.–Mexico border and proximal to it. For the purposes of this study, the border zone was defined as living within 100 kilometers of the U.S.–Mexico border.^21^ Border counties in the U.S.–Mexico region face unique challenges, including higher prevalence of diabetes and obesity, cross-border mobility, and barriers to healthcare access. Prior work in Texas and Arizona border counties have shown higher mortality than in nonborder areas, suggesting that structural disadvantage plays a role.^22^^,^^23^

## METHODS

### Study Design

This retrospective observational study used health records from patients with confirmed SARS-CoV-2 infection across 8 counties in southern NM between June 2020 and December 2022. The authors hypothesize that demographic characteristics and underlying comorbidities are significantly associated with SARS-CoV-2 (COVID-19) outcomes and that the outcomes also vary between border and nonborder counties in southern NM. Data on demographics, comorbidities, and clinical outcomes were extracted with the goal of examining the associations between patient characteristics and COVID-19 outcomes comparing border and nonborder counties in NM.

### Study Sample

The authors analyzed data from COVID-19–positive patients collected by the New Mexico Department of Health (NMDOH) from June 2020 to December 2022. The data set included individuals aged ≥20 years (*n*=93,586). Inclusion criteria were (1) patients with a confirmed SARS-CoV-2–positive test result during the study period and (2) those aged ≥20 years. Exclusion criteria included prisoners and pregnant women. Patient cases were randomly selected from across the state for case investigation, although they were not stratified by county or ZIP code to account for population density. Daily surveyor capacity was variable; about one third of positive cases underwent case investigation during peak periods of the pandemic. The data set covered 8 NM counties—6 in border zone (95.70%) and 2 nonborder (4.30%).

### Measures

Data collection through case investigations was conducted by both NMDOH-employed surveyors and contracted surveyors. Surveyors contacted patients or their representatives and administered oral questionnaires in either English or Spanish, depending on the participant’s preference. The depth and scope of the answers varied on the basis of patient interest, availability, or willingness to respond.

The authors strived to examine as much of the data as possible that straddled the peaks of the national COVID-19 pandemic and some of the time frames during which the virulence was lowered. The dates were selected by the NMDOH and the investigators because this was a period when the most robust information was available. The study investigators were blinded from patient-identifying data by NMDOH before the data were shared with the investigators. Only the raw data were shared with the investigators.

In terms of patient demographic factors, data were collected on age, sex, border status, ethnicity, smoking status, and other medical comorbidities that have been noted to put patients at risk of worse COVID-19 outcomes. Table 1 shows the demographic profile of 93,586 patients with COVID-19: female (54.77%), Hispanic (48.49%), and aged 20–39 years (42.29%). Most lived in border counties (95.70%). Smoking status was categorized as never smokers (11.56%); former smokers (2.82%); current smokers (1.27%); and ever smokers (0.18%), meaning individuals who have smoked at any point in their lives, including both current and former smokers.

**Table 1:** Demographic Characteristics, Comorbidity Conditions, and COVID-19 Patients Outcomes (N=93,586)

Variables	Counts, *n*	Percentages, %
Border counties	89,559	95.70
Nonborder counties	4,027	4.30
Border counties		
Grant	7,918	8.46
Sierra	2,134	2.28
Hidalgo	1,077	1.15
Luna	6,408	6.85
Doña Ana	58,919	62.96
Otero	13,103	14.00
Nonborder counties		
Catron	440	0.47
Socorro	3,587	3.83
Age, years		
20–39	39,574	42.29
40–64	34,612	36.98
≥65	15,166	16.21
Sex		
Male	40,881	43.68
Female	51,254	30.23
Ethnicity		
Hispanic	45,377	48.49
Non-Hispanic	28,288	30.23
Smoking status		
Never smoker	10,820	11.56
Current smoker	1,185	1.27
Former smoker	2,641	2.82
Ever smoker	167	0.18
Comorbidity conditions		
Preexisting conditions		
Yes	11,739	12.54
No	26,761	28.6
Obesity		
Yes	612	0.65
No	3,632	3.88
Hypertension		
Yes	2,553	2.73
No	1,547	1.65
Cardiovascular disease		
Yes	3,234	3.46
No	13,005	13.9
Psychological/psychiatric		
Yes	1,040	1.11
No	3,411	3.64
Chronic lung disease		
Yes	2,551	2.73
No	13,575	14.51
Chronic renal disease		
Yes	819	0.88
No	15,122	16.16
Chronic liver disease		
Yes	368	0.39
No	15,413	16.47
Immunosuppressed disease		
Yes	658	0.70
No	14,299	15.28
Autoimmune disease		
Yes	285	0.3
No	4,013	4.29
Other chronic diseases		
Yes	5,621	6.01
No	10,157	10.85
Health outcomes		
Hospitalized		
Yes	4,445	4.75
No	39,344	42.04
Death from illness		
Yes	1,517	1.62
No	3,893	4.16

*Note*: Table 1 notes the presence or absence of each of the measured comorbid conditions, which included information that was obtained through heath department surveyors. These patient pre-existing conditions before the onset of SARS-CoV-2 infection were used to focus on their impact on hospitalization and mortality. Those patients who are willing to be surveyed (in full or partially) were included in the raw data.

Patients with COVID-19 with comorbid conditions, indicating the presence or absence of each condition, are shown in Table 1. The patient pre-existing conditions prior to COVID-19 infection were used to focus on their impact on hospitalization and mortality.

Table 1 provides counts and percentages for comorbid conditions, pre-existing conditions, and health outcomes. Among the patients with SARS-CoV-2, 4,445 (4.75%) were hospitalized, and 1,517 (1.62%) died. The prevalence of specific comorbidities was as follows: diabetes mellitus (3.42%), obesity (0.65%), hypertension (2.73%), cardiovascular disease (3.46%), psychiatric conditions (1.11%), chronic lung disease (2.73%), chronic renal disease (0.88%), chronic liver disease (0.39%), immunosuppressed disease (0.70%), autoimmune disease (0.30%), and other chronic diseases (6.01%). In addition, 12.54% of patients had 1 or more pre-existing conditions.

### Statistical Analysis

Chi-square tests were conducted to assess the association between COVID-19 outcomes and demographic and comorbidity conditions. Logistic regression models were fitted to investigate the associations with demographic characteristics, each underlying comorbidity condition, and the COVID-19 outcomes. OR and 95% CIs were calculated with the corresponding *p*-values. Missing values were excluded from analyses because the model removed them automatically. The logistic regression models for hospitalization and death are specified as follows:

Hospitalization model:

(1) $$\log{\left(\text{odds}\right)}_{i}=\text{log}\left(\frac{p_{i}}{1-p_{i}}\right)=\beta_{0}+\beta_{1}{\left(\text{Age}\right)}_{i}+\beta_{2}{\left(\text{Sex}\right)}_{i}+\beta_{3}{\left(\text{Hispanic}\;\text{Status}\right)}_{i}+\beta_{4}{\left(\text{Border}\;\text{Status}\right)}_{i}+\beta_{5}{\left(\text{Smoking}\;\text{Status}\right)}_{i}+\beta_{6}{\left(\text{Preexixting}\;\text{conditions}\right)}_{i}$$

(2) $$\log{\left(\text{odds}\right)}_{i}=\text{log}\left(\frac{p_{i}}{1-p_{i}}\right)=\beta_{0}+\beta_{1}{\left(\text{Age}\right)}_{i}+\beta_{2}{\left(\text{Sex}\right)}_{i}+\beta_{3}{\left(\text{Hispanic}\;\text{Status}\right)}_{i}+\beta_{4}{\left(\text{Border}\;\text{Status}\right)}_{i}+\beta_{5}{\left(\text{Smoking}\;\text{Status}\right)}_{i}+\beta_{6}{\left(\text{Each}\;\text{Comobidity}\;\text{Condition}\right)}_{i\;}$$

Death model:

(3) $$\log{\left(\text{odds}\right)}_{i}=\text{log}\left(\frac{p_{i}}{1-p_{i}}\right)=\beta_{0}+\beta_{1}{\left(\text{Age}\right)}_{i}+\beta_{2}{\left(\text{Sex}\right)}_{i}+\beta_{3}{\left(\text{Hispanic}\;\text{Status}\right)}_{i}+\beta_{4}{\left(\text{Border}\;\text{Status}\right)}_{i}+\beta_{5}{\left(\text{Smoking}\;\text{Status}\right)}_{i}+\beta_{6}{\left(\text{Preexixting}\;\text{conditions}\right)}_{i}$$

(4) $$\log{\left(\text{odds}\right)}_{i}=\text{log}\left(\frac{p_{i}}{1-p_{i}}\right)=\beta_{0}+\beta_{1}{\left(\text{Age}\right)}_{i}+\beta_{2}{\left(\text{Sex}\right)}_{i}+\beta_{3}{\left(\text{Hispanic}\;\text{Status}\right)}_{i}+\beta_{4}{\left(\text{Border}\;\text{Status}\right)}_{i}+\beta_{5}{\left(\text{Smoking}\;\text{Status}\right)}_{i}+\beta_{6}{\left(\text{Each}\;\text{Comobidity}\;\text{Condition}\right)}_{i}$$

where $p_{i}$ represents the probability of hospitalization or death for the $i^{th}$ patient.

Statistical analyses were conducted in R (Version 4.2.2) with a significance level of 0.05.

## RESULTS

The analysis of hospitalization using the chi-square test is presented in Table 2. Significant associations were found between demographic factors and hospitalization status. Geographically, nonborder county residents had a higher hospitalization rate (8.53%) than nonhospitalization (3.65%, *p*<0.001). There was a significant association between age group and hospitalization (*p*<0.001), showing the highest rate of hospitalization for individuals aged ≥65 years (53.36%) than for younger age groups. Sex and ethnicity were also associated with hospitalization status (*p*<0.001). Males exhibited a higher hospitalization rate (51.53%) than females (48.47%), and non-Hispanics had an increased share of hospitalized group (43.61%) than of nonhospitalized group (32.30%). In addition, smoking status and hospitalization indicated a significant association (*p*<0.001).

**Table 2:** Characteristics of the Patients With COVID-19, Stratified by Outcomes of Hospitalization and Deaths

Variables	Hospitalized (*n*=4,445)	Not hospitalized (*n*=39,344)	*p*-value	Deaths (*n*=151)	Alive (*n*=3,893)	*p*-value
Border status						
Border	4,066 (91.47)	37,906 (96.35)	**<0.001**	1,406 (92.68)	3,773 (96.92)	**<0.001**
Nonborder	379 (8.53)	1,438 (3.65)		111 (7.32)	120 (3.08)	
Age, years						
20–39	496 (11.22)	17,013 (45.45)	**<0.001**	58 (3.82)	1,625 (43.44)	**<0.001**
40–64	1,566 (35.42)	15,241 (40.72)		362 (23.86)	1,299 (34.72)	
≥65	2,359 (53.36)	5,178 (13.83)		1,097(72.31)	817 (21.84)	
Sex						
Male	2,286 (51.53)	16,854 (43.07)	**<0.001**	867 (57.19)	2,020 (52.35)	**<0.001**
Female	2,150 (48.47)	22,274 (56.93)		649 (42.81)	1,839 (47.65)	
Ethnicity						
Hispanic	2,410 (56.39)	25,434 (67.70)	**<0.001**	848 (56.01)	2,101 (57.70)	0.277
Non-Hispanic	1,864 (43.61)	12,137 (32.30)		666 (43.99)	1,540 (42.30)	
Smoking status						
Never smoker	1,085 (72.09)	9,392 (73.12)	**<0.001**	433 (76.91)	1,516 (79.41)	**<0.001**
Current smoker	53 (3.52)	1,101 (8.57)		13 (2.31)	56 (2.93)	
Former smoker	322 (21.4)	2,240 (17.44)		106 (18.83)	242 (12.68)	
Ever smoker	45 (2.99)	111 (0.86)		11 (1.95)	95 (4.98)	

*Note*: Boldfaces indicate statistical significance on the chi-square tests.

Table 3 describes the results from 2 distinct models: one examining hospitalization model (Model 1) and the other focusing on death model (Model 3). Hospitalization model (Model 1) assesses the ORs with 95% CIs and corresponding *p*-values for various demographic factors and pre-existing conditions. Age is strongly associated with hospitalization risk, indicating an increasing likelihood of hospitalization with older age. Individuals aged 40–64 years exhibit 162% higher odds of hospitalization (OR=2.62; 95% CI=2.18, 3.17; *p*<0.001), whereas those aged ≥65 years show an even more substantial 570% increase (OR=6.70; 95% CI=5.54, 8.14; *p*<0.001) than the age group of 20–39 years. Males have 40% higher odds of hospitalization (OR=1.40; 95% CI=1.24, 1.58; *p*<0.001) than females. Nonborder county residents demonstrate a 157% higher likelihood of hospitalization (OR=2.57; 95% CI=2.03, 3.25; *p*<0.001) than border county residents. Interestingly, current smokers have 67% lower odds of hospitalization (OR=0.33; 95% CI=0.24, 0.45; *p*<0.001), whereas former smokers show a 36% decrease in odds (OR=0.64; 95% CI=0.55, 0.74; *p*<0.001). Conversely, ever smokers exhibit 159% higher odds of hospitalization (OR=2.59; 95% CI=1.73, 3.84; *p*<0.001). Finally, individuals with pre-existing conditions have 387% higher odds of hospitalization (OR=4.87; 95% CI=3.99, 6.00; *p*<0.001) than those without such conditions, highlighting the strong association between pre-existing health issues and increased hospitalization risk.

**Table 3:** Estimated ORs and 95% CIs From the Logistic Regression Model Including Pre-Existing Conditions

	Hospitalization Model 1		Death Model 3	
Variable	OR (95% CI)	*p*-value	OR (95% CI)	*p*-value
Age (ref^a^: 20–39)				
40–64	2.62 (2.18, 3.17)	**<0.001**	5.95 (3.50, 10.86)	**<0.001**
≥65	6.70 (5.54, 8.14)	**<0.001**	17.48 (10.37, 31.70)	**<0.001**
Sex (ref: female)				
Male	1.40 (1.24, 1.58)	**<0.001**	1.86 (1.50, 2.30)	**<0.001**
Hispanic status (ref: non-Hispanic)				
Hispanic	1.12 (0.99, 1.28)	0.072	1.60 (1.28, 1.99)	**<0.001**
Border status (ref: border)				
Nonborder	2.57 (2.03, 3.25)	**<0.001**	3.10 (2.06, 4.68)	**<0.001**
Smoking status (ref: never smoker)				
Current smoker	0.33 (0.24, 0.45)	**<0.001**	1.74 (0.81, 3.50)	0.135
Former smoker	0.64 (0.55, 0.74)	**<0.001**	1.22 (0.92, 1.63)	0.170
Ever smoker	2.59 (1.73, 3.84)	**<0.001**	0.64 (0.31, 1.22)	0.200
Pre-existing condition (ref: no)				
Yes	4.87 (3.99, 6.00)	**<0.001**	2.92 (1.72, 5.33)	**<0.001**

*Note*: Boldfaces indicate statistical significance.

a ref: ORs are reported relative to the designated reference category.

Table 4 represents the hospitalization model (Model 2), which assesses the impact of various comorbidities on the odds of hospitalization, as indicated by ORs with 95% CIs and corresponding *p*-values. The presence of comorbidities in patients with SARS-CoV-2 significantly increases the odds of hospitalization. Notably, diabetes mellitus is associated with 221% higher odds (OR=3.21; 95% CI=2.81, 3.65), whereas obesity shows 104% higher odds (OR=2.04; 95% CI=1.57, 2.66). Hypertension is linked to a 154% increase in hospitalization odds (OR=2.54; 95% CI=2.11, 3.07), and cardiovascular disease presents 105% higher odds (OR=2.05; 95% CI=1.79, 2.35). Chronic lung disease and chronic liver disease are associated with 109% (OR=2.09; 95% CI=1.80, 2.43) and 222% higher odds (OR=3.22; 95% CI=2.35, 4.38), respectively. Chronic renal disease significantly increases hospitalization odds by 498% (OR=5.98; 95% CI=4.89, 7.31), whereas immunosuppression disease shows 185% higher odds (OR=2.85; 95% CI=2.28, 3.55). Autoimmune disease is linked to 80% higher odds (OR=1.80; 95% CI=1.25, 2.58), and other chronic diseases present 414% higher odds of hospitalization (OR=5.14; 95% CI=4.47, 5.92) (all *p*<0.001). Conversely, psychiatric disorders do not significantly impact hospitalization risk (OR=1.14; 95% CI=0.94, 1.39; *p*=0.186).

**Table 4:** Estimated ORs and Their 95% CIs for Comorbidity Using Logistic Regression Models

	Hospitalization Model 2		Death Model 4	
Variable	OR (95% CI)	*p*-value	OR (95% CI)	*p*-value
Diabetes mellitus (ref: no)				
Yes	**3.21 (2.81, 3.65)**	**<0.001**	1.78 (1.42, 2.23)	**<0.001**
Obesity (ref: no)				
Yes	**2.04 (1.57, 2.66)**	**<0.001**	1.70 (1.13, 2.53)	**<0.001**
Hypertension (ref: no)				
Yes	2.54 (2.11, 3.07)	**<0.001**	**2.22 (1.73, 2.85)**	**<0.001**
Cardiovascular disease (ref: no)				
Yes	**2.05 (1.79, 2.35)**	**<0.001**	**3.00 (2.36, 3.82)**	**<0.001**
Psychiatric (ref: no)				
Yes	**1.14 (0.94, 1.39)**	**0.186**	**0.60 (0.46, 0.79)**	**<0.001**
Chronic lung disease (ref: no)				
Yes	**2.09 (1.80, 2.43)**	**<0.001**	**1.37 (1.05, 1.78)**	**<0.001**
Chronic renal disease (ref: no)				
Yes	**5.98 (4.89, 7.31)**	**<0.001**	**3.07 (2.26, 4.18)**	**<0.001**
Chronic liver disease (ref: no)				
Yes	**3.22 (2.35, 4.38)**	**<0.001**	**2.37 (1.31, 4.21)**	**<0.001**
Immunosuppressed disease (ref: no)				
Yes	**2.85 (2.28, 3.55)**	**<0.001**	**3.36 (2.10, 5.35)**	**<0.001**
Autoimmune disease (ref: no)				
Yes	1.80 (1.25, 2.58)	**<0.001**	**2.63 (1.46, 4.63)**	**<0.001**
Other chronic diseases (ref: no)				
Yes	5.14 (4.47, 5.92)	**<0.001**	**1.94 (1.48, 2.55)**	**<0.001**

*Note*: Boldfaces indicate statistical significance.

ORs are reported relative to the designated reference category.

The results of death outcome are presented in Table 2. The analysis examined the impact of border status, age, sex, ethnicity/race, and smoking status on the death outcome. Significant associations were found for border status, age, sex, and smoking status (all *p*<0.001). However, race/ethnicity did not show a statistically significant association with mortality risk (*p*=0.277). In the death model (Model 3) of Table 4, age is identified as a significant risk factor for mortality. Individuals aged 40–64 years exhibit 495% higher odds of death (OR=5.95; 95% CI=3.50, 10.86; *p*<0.001), whereas those aged ≥65 years show an even more substantial 1,648% increase (OR=17.48; 95% CI=10.37, 31.70; *p*<0.001) than those aged 20–39 years. Males are associated with 86% higher odds of death (OR=1.86; 95% CI=1.50, 2.30; *p*<0.001) than females, and Hispanic individuals have 60% higher odds of death (OR=1.60; 95% CI=1.28, 1.99; *p*<0.001) than non-Hispanics. Nonborder residents face 210% increased odds of death (OR=3.10; 95% CI=2.06, 4.68; *p*<0.001) than border county residents. Whereas current smokers show a 74% increased mortality risk (OR=1.74; 95% CI=0.81, 3.50; *p*=0.135), and former smokers exhibit a 22% higher risk (OR=1.22; 95% CI=0.92, 1.63; *p*=0.170) than never smokers, ever smokers demonstrate a 36% lower risk (OR=0.64; 95% CI=0.31, 1.22; *p*=0.200). However, smoking status is not statistically significant for mortality. In addition, individuals with pre-existing conditions show 192% higher odds of death (OR=2.92; 95% CI=1.72, 5.33; *p*<0.001).

Table 4 represents the death model (Model 4), which assesses the impact of various comorbidities on the odds of deaths, as indicated by ORs with 95% CIs and corresponding *p*-values. The presence of comorbidities in patients with SARS-CoV-2 is significantly associated with increased odds of mortality. Specifically, diabetes mellitus is linked to 78% higher mortality (OR=1.78; 95% CI=1.42, 2.23), obesity is linked to 70% higher mortality (OR=1.70; 95% CI=1.13, 2.53), and hypertension is linked to 122% higher mortality (OR=2.22; 95% CI=1.73, 2.85). In addition, cardiovascular disease is associated with 200% higher mortality (OR=3.00; 95% CI=2.36, 3.82), chronic lung disease is associated with 37% higher mortality (OR=1.37; 95% CI=1.05, 1.78), and chronic renal disease is associated with 207% higher mortality (OR=3.07; 95% CI=2.26, 4.18). Chronic liver disease shows 137% higher mortality (OR=2.37; 95% CI=1.31, 4.21), immunosuppression disease presents 236% higher mortality (OR=3.36; 95% CI=2.10, 5.35), and autoimmune disease is associated with 163% higher mortality (OR=2.63; 95% CI=1.46, 4.63). Other chronic diseases also demonstrate 95% higher mortality (OR=1.95; 95% CI=1.48, 2.55) (all *p*<0.001). In contrast, psychiatric disorders are associated with 40% reduced odds of mortality (OR=0.60; 95% CI=0.46, 0.79; *p*<0.001).

## DISCUSSION

The results identified several demographic and clinical predictors of COVID-19 outcomes in southern NM. Male patients had 40% higher odds of hospitalization and 86% higher odds of death than females. This is consistent with higher risks observed among males in prior studies.^10^^,^^24^ Age was the strongest predictor, with patients aged ≥65 years facing nearly sevenfold greater odds of hospitalization and more than 17-fold greater odds of death than adults aged 20–39 years, a pattern also reported globally.^9^^,^^12^^,^^14^ The authors also observed significant geographic disparities: nonborder county residents had more than twice the odds of hospitalization and 3 times the odds of death than border residents, aligning with border health research highlighting inequities in care access.^22^^,^^23^ Most of the conclusions presented in this paper are supported by the broader literature except smoking, psychiatric disorders, and ethnicity. Although Hispanic ethnicity (majority of state dwellers) was not a significant predictor of hospitalization, Hispanic patients experienced 60% higher odds of death than non-Hispanic patients, consistent with studies documenting disproportionate mortality in Hispanic populations.^24^, ^25^, ^26^ Similar disparities in hospitalization have also been documented, particularly at the intersection of race, ethnicity, and income, where Black and Latino patients experienced disproportionately higher hospitalization risk, which was not seen in Hispanic ethnicity in the study and its link hospitalization noted in other studies.^27^^,^^28^ The authors postulate that proximity to larger cities could play an important role in their state. This could be explored further with data on health utilization and resource allocation. Finally, pre-existing conditions markedly increased risk, with nearly fivefold higher odds of hospitalization and almost threefold higher odds of death than in those without comorbidities, reinforcing global evidence that diabetes, hypertension, and other chronic illnesses amplify COVID-19 severity.^7^^,^^8^^,^^11^^,^^12^

The findings also align with international evidence from China, Italy, the Middle East, and sub-Saharan Africa. Male sex and older age have consistently been reported as strong predictors of severe outcomes in multiple regions.^9^^,^^12^^,^^14^^,^^18^^,^^19^ Prior U.S. and global studies have highlighted disproportionate mortality among Hispanic and Latino populations as well as among marginalized ethnic groups, underscoring structural inequities in health care.^24^, ^25^, ^26^^,^^28^ The strong associations between comorbidities, particularly diabetes, hypertension, and cardiovascular disease, and adverse outcomes are supported by extensive literature across Latin America, the Middle East, and Africa.^7^^,^^8^^,^^17^, ^18^, ^19^, ^20^ The geographic finding that nonborder residents were at greater risk differs from some prior work and highlights the importance of local healthcare access and utilization patterns.^22^^,^^23^ By analyzing the findings in a broader global context, the authors demonstrate that although certain risk factors (such as age and comorbidities) are consistent across countries, the role of ethnicity and geography can differ depending on local health systems and their responses, social determinants, and structural inequities. This observation is consistent with systematic reviews highlighting racial, ethnic, and socioeconomic disparities in COVID-19 outcomes across multiple regions worldwide.^29^

These results have important implications for public health and healthcare delivery in NM. Targeted allocation of healthcare resources to nonborder areas could address disparities in access and outcomes. Prioritizing early education and preventive interventions may reduce the burden of comorbidities that amplify COVID-19 risk. Increasing community awareness of high-risk factors and strengthening bilingual health communication in English and Spanish can improve outreach to diverse populations. Strengthening bilingual health communication is critical for reaching Hispanic populations and should be best practice. Targeted vaccination and early treatment strategies for vulnerable groups, combined with the use of community health workers for outreach and education, can enhance healthcare delivery and reduce inequities in both border and nonborder counties. In addition, the authors believe that community health workers for outreach is supported by literature and could be a key strategy for both border and nonborder counties. Finally, structurally inequities such as insurance coverage, transportation, healthcare access, and trust in the medical system should all be investigated as it relates to comorbidities.

The results of this study carry broader implications for clinical care, public health, and future research. Clinically, identifying older adults, males, and patients with chronic comorbidities as high-risk groups underscores the importance of early intervention and tailored care strategies. At the public health level, the observed disparities between border and nonborder counties highlight the importance of equitable resource distribution, community health worker engagement, and bilingual health communication to reach diverse populations. Academically, the study contributes to the growing body of literature on COVID-19 disparities and provides a framework for comparative research across different regions and populations. Looking ahead, these findings emphasize the need for preparedness strategies that integrate demographic and comorbidity risk factors into pandemic response planning, while also addressing structural and geographic inequities in healthcare delivery.

### Limitations

Several limitations were encountered during this research study. Research has shown disparities in COVID hospitalizations on the basis of race, ethnicity, and income.^27^^,^^28^ Unfortunately, the data lacked information about income. Future research should aim to further stratify the data to assess the impact of household income on hospitalization and mortality. In addition, the sampling size for each county was not proportional to its actual population size, indicating that the health department may have placed less assets dedicated to these areas, or the COVID 19 cases were under tested or reported. This may have led to some potential bias and reduced the generalizability of the findings. Future studies should employ stratified sampling proportional to county population size to ensure adequate representation across regions. Alternatively, survey weights can be applied so that the sample reflects the underlying population distribution by age, sex, ethnicity, and county. Only patients who were willing to be surveyed (fully or partially) were included (nonresponse bias). In some counties, the authors suspected that patients were not getting tested, being managed at home, and not taken to medical facilities, potentially introducing selection bias. In addition, some counties may have had lower testing rates, with patients managed at home and not taken to medical facilities, thus underreporting cases and outcomes, particularly in less-resourced counties.

Furthermore, the vaccination status of participants was not tracked in the data nor accounted for once the first available vaccine became available during this time frame, which could impact the interpretation of disease outcomes and their association with vaccination status (confounding bias). Finally, the disease categories as defined by the NMDOH could be considered too broad, and a more refined categorization could have provided deeper insights into specific health conditions. Addressing these limitations is crucial for enhancing the robustness and accuracy of future research in this area.

## CONCLUSIONS

This study aimed to identify the demographic and comorbidity factors associated with hospitalization and mortality among patients with COVID-19 in southern NM. The authors found that older age, male patients, residents in nonborder counties, smoking status, and the presence of pre-existing medical conditions were strongly associated with an increased risk of hospitalization and death. In particular, comorbidities such as chronic renal disease, chronic liver disease, diabetes, hypertension, and cardiovascular conditions showed strong associations with hospitalization and mortality. Although ethnicity was not a significant factor in the hospitalization model, it was associated with mortality risk.

By highlighting these key risk factors, the study enhances the understanding of vulnerable populations within the community. These findings provide valuable evidence for guiding public health strategies, particularly in clinical management and resource allocation for high-risk groups such as older adults and individuals with multiple comorbidities and ultimately aiming to improve outcomes during future infectious disease outbreaks.
